# Comparison of Monopolar Electrosurgical Conization and the Loop Electrosurgical Excision Procedure in the Management of High-Grade Squamous Intraepithelial Lesion

**DOI:** 10.3389/fsurg.2021.721545

**Published:** 2021-09-20

**Authors:** Jun Ding, Haiou Xu, Lihua Xia, Shanshan Cao, Qing Wu

**Affiliations:** ^1^Reproductive Medicine Center, Department of Gynecology, Zhejiang Provincial People's Hospital, Affiliated People's Hospital, Hangzhou Medical College, Hangzhou, China; ^2^Hangzhou Women's Hospital, Hangzhou, China; ^3^Tiantai People's Hospital of Zhejiang Province, Zhejiang, China

**Keywords:** high-grade squamous intraepithelial lesion, LEEP, HPV, pathology, cervical conization

## Abstract

**Objectives:** To compare the performance and outcomes of monopolar electrosurgical conization (MESC) or the loop electrosurgical excision procedure (LEEP) in the treatment of high-grade squamous intraepithelial lesion (HSIL).

**Methods:** This retrospective study included 554 patients diagnosed with HSIL through biopsy. The study used either LEEP or MESC for cervical conization. Additionally, the medical records of these patients, including the basic information, status of the excision margin, cone depth, cone width, fragmentation, complication, and the results of a 6-month follow-up after conization, were reviewed.

**Results:** Compared to MESC, LEEP had a significantly higher rate of positive endocervical margin (3.77 vs. 8.65%; *p* = 0.018), burn injury of the margin (4.90 vs. 10.38%; *p* = 0.016) and a lower rate of adequate cone depth (83.40 vs. 89.62%; *p* = 0.034). In addition, LEEP was significantly more likely to cause fragmentation (*p* = 0.000). There was, however, no significant difference in the rate of abnormal cervical cytology and positive high-risk HPV (hrHPV) between these two groups, 6 months after cervical conization.

**Conclusion:** Both LEEP and MESC appeared to be equally effective in the clinical treatment of HSIL. Nonetheless, MESC resulted in a better pathological outcome with regard to the status of the margin, tissue fragmentation, and cone depth.

## Introduction

Based on recommendations by the American Society for Colposcopy and Cervical Pathology (ASCCP), cervical excision is mainly used to treat high-grade squamous intraepithelial lesion (HSIL), which might progress to invasive cervical cancer if left untreated (1). Currently, one of the most common excisional strategies for treating HSIL is the loop electrosurgical excision procedure (LEEP), which enables deep excision of the cervical transformation zone (TZ) with minimal damage. In addition, LEEP is associated with several advantages, including a shorter operative time, ease of performance, and low cost (2, 3). However, limited specimen volume, high risk of margin involvement, and tissue fragmentation are the common problems associated with LEEP when utilized in HISL or more severe cervical lesions (4).

In our center, it was proposed that cervical electrosurgical conization using the monopolar scalpel could avoid tissue fragmentation. However, there were still doubts about complication, the status of the margin and other pathological outcomes following monopolar electrosurgical conization (MESC). Therefore, the present study aimed to compare the performance and outcome of LEEP or MESC in the treatment of HSIL. Moreover, the study conducted cervical screening 6 months after LEEP or MESC for HSIL.

## Materials and Methods

This retrospective study was approved by the ethics committee of Zhejiang Provincial People's Hospital (NO.2021QT047). The study recruited patients who had undergone colposcopy-guided biopsy for HSIL, had received LEEP or MESC for the treatment of histologically proven HSIL, and accepted the 6-month follow-up period. The patients were recruited from January 2017 to December 2019 in the Department of Gynecology, Zhejiang Provincial People's Hospital. Detailed surgical and pathological data was collected from electronic medical records.

In addition, LEEP or MESC was performed in women diagnosed with HSIL through cervical biopsies. However, the study excluded women without HSIL in the cervical conization specimens, those diagnosed with invasive cancer, or individuals lacking any follow-up record.

### Surgical Procedures

The same gynecologist group performed either LEEP or MESC. Notably, LEEP was performed with a loop electrode and an electrosurgical unit in a blended mode consisting of a 50-W cutting current and 30-W coagulation. On the other hand, MESC was conducted using a monopolar electrical scalpel commonly used for open surgeries. During the incision procedure, MESC applied an electrocision of 40 W rather than electrocoagulation to avoid charring the margin of the specimen. However, when some bleeding was noticed, electrocoagulation was performed until hemostasis was verified.

While preparing for conization, the Lugol's iodine solution was applied to distinguish the extent of cervical lesions under colposcopy and predetermine the circumference and width of excision. After visualization of the cervix and the squamocolumnar junction, the TZ was assessed in its native condition (Type 1: TZ fully visible; Type 2: TZ partly visible; Type 3: TZ not visible). Additionally, the length of excision depended on the TZ. Herein, 3–5 mm ectocervical resection margins were typically used. If the TZ was fully observed, 1 cm of the cervix was removed. However, if the TZ was Type 2, ~1.5 cm of specimen was removed, and if the TZ was Type 3, approximately more than 2 cm of specimen was removed. The specimen was then marked at the 12 o'clock position after cervical conization. Moreover, each specimen was measured to determine its length and width, before fixation. All the histopathology reports were confirmed by two pathologists, including one senior pathologist.

### Pathological Assessment and Follow-Up

The medical records of these patients were reviewed, and the following information was recorded; age, body mass index (BMI), parity, use of oral contraceptive pills (OCP), smoking, type of the TZ, pathology of the specimen margins, endocervical involvement, ectocervical involvement, burn injury of the margin, cone depth, cone width, fragmentation, procedure time, intraoperative blood loss, complication, and the result of follow-up.

The operative time was determined from the insertion of the blade to the confirmation of hemostasis. The margins of the specimens were grouped into the following categories: endocervical margin (inner side of the incision and the deep margin), ectocervical margin, and burn injury of the margin. In addition, the resection margin was considered negative if abnormal cells were not found on the margin of the cone specimen or positive if abnormal cells were identified on the margin of the cone specimen. An adequate cone depth was identified through the following criteria: if the TZ was fully observed, 1 cm of the cervix was removed; if the TZ was Type 2, approximately a 1.5 cm specimen was removed; while if the TZ was Type 3, approximately more than 2 cm of specimen was removed. Moreover, an adequate cone width was identified as a margin of 3–5 mm outside the TZ. Intraoperative and postoperative complications were noted if they occurred within 15 days after conization (e.g., vaginal burn, adjacent organ burn, postoperative bleeding, cervical stenosis, and local cervical or uterine infection).

Furthermore, follow-up was recommended and scheduled at 6, 12, and 24 months after cervical conization. Follow-up included the detection for HPV through Cervista® (Hologic, Bedford, MA, USA) and ThinPrep liquid-based cytology (Hologic, Inc., San Diego, CA, USA). Cervical cytology was reported according to the 2001 Bethesda System. Notably, a cytology report of atypical squamous cells of undetermined significance and above was considered abnormal. Finally, a colposcopic evaluation was performed if the results were abnormal.

### Statistical Analysis

Data on continuous variables was reported as medians and ranges whereas categorical data was presented as counts and percentages. Before conducting the two-sample *t*-test, data was confirmed to be normally distributed. In addition, continuous variables as age, BMI, and procedure time were analyzed using the two-sample *t*-test. On the other hand, the Chi-square test was used to compare categorical data such as status of the margin, fragmentation, and complication. Moreover, the Fisher-exact test was applied for categorical data if *n* < 5. The SPSS 17.0 software (SPSS Inc., Chicago, IL, USA) was used to conduct the statistical analyses. Additionally, all statistical tests were two tailed and a *p* < 0.05 was considered to be statistically significant.

## Results

In this study, 56 patients only received the HPV test and ThinPrep liquid-based cytology at 12 or 24 months, without follow-up at the 6th month after cervical conization. They were consequently excluded from further analysis. However, 554 patients met the inclusion criteria of the study. The patients were divided into two groups according to the method of cervical conization, namely; the LEEP and MESC categories. The patient characteristics according to study allocation are shown in Table 1. The findings showed that there were no significant differences in age, BMI, parity, use of oral contraceptive pills, alcohol abuse, and cigarette smoking, between the two groups (*p* > 0.05). Moreover, no significant differences were obtained with respect to the distribution of TZ, between these two groups (see Table 1).

**Table 1:** Characteristics of patients with HSIL of the cervix.

	**MESC (** ***n =*** **265)**	**LEEP (** ***n =*** **289)**	***P***
Age (y)	34.3 (20–59)	35.4 (22–64)	0.557
BMI (kg/m^2^)	23.3 (19.1–35.3)	24.2 (19.3–35.2)	0.568
Parity (*n*)	213	221	0.302
Smoking (*n*)	45	52	0.823
Alcohol abuse (*n*)	36	42	0.328
OCP (*n*)	18	21	0.869
**Type of transformation zone**			
Type1 (*n*)	120	115	0.198
Type2 (*n*)	56	65	0.758
Type3 (*n*)	89	109	0.330

Table 2 shows a comparison of the outcomes in women assigned to both study groups. Specifically, the study compared the status of the resection margin, cone dimensions (length and width), fragmentation of the specimens, procedure time, intraoperative blood loss and complications.

**Table 2:** Comparison of the outcomes of HISL treated through LEEP or MESC.

	**MESC (** ***n =*** **265)**	**LEEP (** ***n =*** **289)**	***P***
Status of margin			
Positive endocervical margin (*n*)	10	25	0.018
Positive ectocervical margin (*n*)	12	15	0.718
Burn injury of margin (*n*)	13	30	0.016
Adequate Cone depth (*n*)	221	259	0.034
Adequate Cone width(*n*)	256	281	0.806
No. of fragments			
=1 (*n*)	213	150	0.00
≥1 (*n*)	52	139	
Procedure time (min)	28.4 (15–35)	18.6 (15–25)	0.022
Intraoperative blood loss (ml)	27.3 (5–80)	15.6 (5–20)	0.038
Complications			
Intraoperative (*n*)	5	3	0.489
Postoperative (*n*)	27	36	0.401

In Table 2, the study compared the characteristics of specimens obtained from these two groups. The results showed that there were no significant differences in the rates of the positive ectocervical margin (4.53 vs. 5.19%; *p* = 0.718) between the two groups. However, the LEEP group had a significantly higher rate of positive endocervical margin (3.77 vs. 8.65%; *p* = 0.018) and burn injury of the margin (4.90 vs. 10.38%; *p* = 0.016). In addition, LEEP was significantly more likely to cause fragmentation (*p* = 0.000) than MESC. The findings also revealed that the rate of adequate cone depth was significantly less in LEPP than in MESC (83.40 vs. 89.62%; *p* = 0.034). Moreover, both the intraoperative blood loss and procedure time were short in these two groups, although the figures were higher in MESC than in LEEP (*p* = 0.022; *p* = 0.038).

The study also observed eight intraoperative and 63 postoperative complications. Nonetheless, the rate of intraoperative and postoperative complications was not significantly different between these two study groups. Additionally, the rate of abnormal cervical cytology and positive high-risk HPV (hrHPV), at 6 months after cervical conization were slightly higher in the LEEP group than in the MESC category, although the differences were not significant (13.96 vs. 10.38%; *p* = 0.240; 15.47 vs. 11.07%; *p* = 0.133, Table 3).

**Table 3:** The cytology and the HPV test at a 6-month follow-up visit after LEEP or MESC.

	**MESC (** ***n =*** **265)**	**LEEP (** ***n =*** **289)**	***P***
Abnormal cervical cytology (*n*)	30(11.32%)	41 (14.19%)	0.313
Positive high-risk human papilloma virus (hrHPV) testing (*n*)	41 (15.47%)	53 (18.34%)	0.369

## Discussion

In 2014, the age-standardized incidence and mortality rates of cervical cancer in China were 11.6 and 3.1/100,000, respectively, which were relatively higher than those from developed countries (5). Therefore, management of cervical cancer precursors such as HSIL is very important in preventing the progression of the malignancy. Although LEEP is an effective and appropriate strategy for the treatment of HSIL, some patients experience persistent HSIL, recurrence or even progressive lesions after LEEP (6, 7). Moreover, while LEEP is easy to complete, it is argued that the tissue margins in a LEEP biopsy may show significant thermal artifacts, which can interfere with the pathological assessment of biopsy margins (8). LEEP is also associated with higher rates of the fragmentation of cervical excisional biopsy specimens, which is associated with a higher risk of positive margins and indeterminate margins at time of the procedure, compared to unfragmented specimens (4, 9). In addition, incomplete excision is associated with 5 times the risk of post-treatment lesions and 6 times the rate of subsequent HSIL, as compared to complete excision (10).

Historically, cold knife conization was used as a primary method of cervical excision to treat squamous intraepithelial lesions. However, alternative methods, such as electrosurgical scalpel conization and LEEP are equally effective in clinical treatment (11, 12). To the best of our knowledge, this is the first study to compare the performance of LEEP with that of MESC. The results showed that the rate of abnormal cervical cytology and positive hrHPV in MESC were not significantly different from those in LEEP (13.96 vs. 10.38%; *p* = 0.240; 15.47 vs. 11.07%; *p* = 0.133). Moreover, the rate of intraoperative and postoperative complications in LEEP were similar to those in MESC (*p* = 0.489; *P* = 0.401).

However, the findings showed that LEEP had a significantly higher rate of a positive endocervical margin (3.77 vs. 8.65%; *p* = 0.018) and burn injury of the margin (4.90 vs. 10.38%; *p* = 0.016). Additionally, LEEP was significantly more likely to cause fragmentation than MESC (*p* = 0.000), and the rate of adequate cone depth in LEEP was significantly less than that in MESC (83.40 vs. 89.62%; *p* = 0.034). The study also showed that LEEP caused higher rates of specimen fragmentation, a higher rate of positive endocervical margin and burn injury of the margin, and a lower rate of adequate cone depth. Moreover, the results revealed that MESC for HSIL resulted in better cones, suggesting that the MESC protocol was a better alternative to LEEP. Notably, the monopolar electrosurgical equipment exists widely, and the manipulations can easily be performed without special instruments and training, which may shorten the learning curve for conization.

Although the study uncovered some novel findings, it had a number of limitations. First, this was a retrospective study that was conducted in a single medical center. In addition, many patients could not complete a follow-up of 2 years, which limited the sample size and long-term results of the study. A more extensive follow-up of this study and a well-designed randomized clinical trial is therefore required to compare the pathological description and clinical outcomes. Moreover, the risk of pregnancy-related and long-term complications were not evaluated.

## Conclusions

In summary, both LEEP and MESC appeared equally effective in the clinical treatment of HSIL. However, MESC resulted in a better pathological description with regard to the status of the margin, tissue fragmentation, and cone depth. The MESC protocol may therefore be a better alternative to LEEP in the treatment of HSIL.

## Data Availability Statement

The data analyzed in this study is subject to the following licenses/restrictions: The data of study are not publicly available due to ethical and legal restrictions. However, upon request, data may be available from the corresponding author on reasonable request. Requests to access these datasets should be directed to okwq31@163.com.

## Ethics Statement

The studies involving human participants were reviewed and approved by the Institutional Ethics Committee of Zhejiang Provincial People's Hospital, China. The patients/participants provided their written informed consent to participate in this study.

## Author Contributions

QW, JD, and LX not only conceived and designed the study but also participated in the drafting and writing of the manuscript. They also supervised the study and critically revised the manuscript. QW, SC, and JD collected the clinical data. QW and HX were responsible for drafting and writing the manuscript and conducting statistical analyses. All authors substantially contributed to the revision of the manuscript.

## Funding

This study was supported by the Zhejiang Chinese Traditional Medicine Scientific Research Fund Project (2021ZB025) and the Health Science and Technology Program of Zhejiang Province (2021KY504). The funders had no role in the study design, data collection and analysis, decision to publish, or preparation of the manuscript.

## Conflict of Interest

The authors declare that the research was conducted in the absence of any commercial or financial relationships that could be construed as a potential conflict of interest.

## Publisher's Note

All claims expressed in this article are solely those of the authors and do not necessarily represent those of their affiliated organizations, or those of the publisher, the editors and the reviewers. Any product that may be evaluated in this article, or claim that may be made by its manufacturer, is not guaranteed or endorsed by the publisher.

## Abbreviations

LEEP: Loop electrosurgical excision procedure
HSIL: High-grade squamous intraepithelial lesion
TZ: Transformation zone
BMI: Body mass index
OCP: Oral contraceptive pills
hrHPV: High-risk HPV.
